# Dynamic Compressive Behavior and Internal Damage Evolution of Polypropylene Fiber-Reinforced Alkali-Activated Slag Geopolymer Mortar

**DOI:** 10.3390/ma19204263

**Published:** 2026-10-09

**Authors:** Binghui Cui, Yanzhu Liu, Liang Wang

**Affiliations:** 1 School of Management Engineering, Qingdao University of Technology, Qingdao 266500, China; 901020230133@qut.edu.cn; 2 Engineering Research Center , Ministry of Education for Underground Mining Engineering, Anhui University of Science and Technology, Huainan 232001, China; 3 School of Civil Engineering and Architecture, Anhui University of Science and Technology, Huainan 232001, China

**Keywords:** geopolymer mortar, polypropylene fiber, alkali-activated slag, dynamic impact resistance, internal damage evolution

## Abstract

**Highlights:**

• Polypropylene fiber (PPF) improves dynamic impact resistance of alkali-activated slag geopolymer mortar.
• At 1.0 vol% PPF, the dynamic increase factor reaches 2.129 and impact toughness rises by 99.3%.
• Dynamic compressive strength, DIF, and ultimate toughness increase with strain rate (50 s^−1^–130 s^−1^) and PPF content.
• CT analysis shows higher PPF content suppresses large internal voids: 31 voids (>4 mm^3^) at 0.6% versus 2–5 at 0.8–1.0%.
• Fiber bridging and pull-out consume impact energy and inhibit crack initiation and propagation.

**Abstract:**

Ordinary Portland cement (OPC) production is energy- and carbon-intensive, which has motivated the development of more sustainable alternatives such as alkali-activated materials, including granulated blast furnace slag (GBFS) geopolymer mortar. Geopolymer mortar, however, is brittle, has low tensile strength and poor crack resistance, and its damage evolution under dynamic impact remains insufficiently understood. Here we show that polypropylene fiber (PPF) reinforcement substantially improves the dynamic impact resistance of GBFS-based geopolymer mortar with only a limited effect on static compressive strength. Mortars with PPF volume contents of 0%, 0.2%, 0.4%, 0.6%, 0.8%, and 1.0% were prepared at a water-to-binder ratio of 0.55. Flowability, static compressive strength, and dynamic mechanical properties were evaluated using a split Hopkinson pressure bar at strain rates of 50 s^−1^–130 s^−1^, and failure morphology was examined through SEM and CT analysis. Increasing PPF content reduced flowability from 253 mm to 224 mm at 1.0% but had little effect on static strength. Dynamic compressive strength, dynamic increase factor (DIF), and ultimate toughness increased with both strain rate and fiber content. The 1.0% PPF mortar reached a maximum DIF of 2.129 and a 99.3% increase in impact toughness. CT analysis further showed that PPF suppressed the formation of large internal voids: approximately 31, 2, and 5 voids larger than 4 mm^3^ were detected in the 0.6%, 0.8%, and 1.0% PPF specimens, respectively. The improvement is attributed to fiber bridging and pull-out, which consume impact energy and inhibit crack initiation and propagation. These results indicate that, within the tested range, a PPF content of 1.0% is effective for impact-resistant geopolymer mortar.

## 1. Introduction

OPC remains the most common binder in construction because of its outstanding strength and durability. However, the production process of OPC is associated with high energy consumption and significant environmental impact. Consequently, there has been growing interest in alternative materials that are environmentally friendly and require lower energy consumption to replace OPC [1,2]. Geopolymer is produced through the reaction of alkali activators (such as NaOH, KOH, and Na_2_SiO_3_) with silicon-rich aluminum inorganic minerals, including fly ash, metakaolin, volcanic ash, and slag [3,4]. This reaction results in the formation of a three-dimensional network of AlO_4_ and SiO_4_ inorganic binder materials [5].

At present, the rapid development of industry has led to a significant problem regarding the inability to effectively utilize industrial waste, resulting in a serious waste of resources. Geopolymer concrete offers a solution by enabling the secondary use of certain types of industrial solid waste. The production of geopolymer concrete can reduce energy consumption by 60% and decrease CO_2_ emissions by 80% [6,7]. The emergence of geopolymer technology is expected to address the conflict between infrastructure development and environmental protection, positively impacting environmental sustainability and aligning with sustainable development strategies [1]. Consequently, many scholars consider alkali-activated materials to be a promising alternative to Portland cement in the future.

Although geopolymer concrete offers advantages such as early strength, rapid setting, dense structure, and resistance to acid and alkali corrosion [1], these characteristics have not led to its widespread adoption in the construction industry. The primary reasons for this limited use are the challenges associated with geopolymer, including shrinkage, low tensile strength, susceptibility to cracking, and poor toughness, which hinder its large-scale application [8]. In the previous engineering application process, considering the deficiencies associated with OPC, Afroughsabet et al. [9] conducted extensive experimental studies. The results showed that the incorporation of fibers in Portland concrete can improve the bending and tensile properties, ductility, toughness, and durability. Recently, Zheng et al. [10] used steel fibers to reinforce the cement concrete. The results showed that these bridging fibers can enhance compressive strength, tensile strength, toughness, impact resistance, water permeability, and reduced shrinkage and cracking. Katkhuda and Shatarat [11] examined the mechanical properties of basalt fiber-reinforced recycled aggregate concrete (BFRC) mixtures, indicating that both tensile and flexural strength were enhanced with basalt fiber at an optimal content of 1% by volume in the mix. Numerous studies have explored the effects of various fibers on ordinary Portland concrete, and the theoretical framework regarding the role of fibers in improving concrete performance has become well established. Given some of the limitations similar to those of OPC, several researchers propose that geopolymer can also benefit from the incorporation of fibers to enhance its performance [12,13].

PPF is a thermoplastic addition polymer composed of propylene monomers. It is widely utilized in the building materials sector due to its low density, high modulus of elasticity, exceptional strength, abundant raw materials, and low manufacturing costs. Parameshwar Hiremath and Subhash C. Yaragal [14] investigated the mechanical properties of PPF when incorporated into reactive powder concrete at varying volumes. The findings indicated that PPF maintained good durability even at high temperatures. Zhu et al. [15] prepared specimens of PPF recycled concrete in different strength grades (C25, C35, and C45). The results demonstrated that PPF significantly enhanced the compressive strength, tensile strength under cracking, and crack resistance of RAC. Shi et al. [16] used a three-point bending test to assess the bending resistance of polyvinyl alcohol-PPF-toughened foamed concrete, examining the bending properties of the specimens in terms of load–deflection curves, failure modes, toughness, and toughening mechanisms. The results indicated that PPF contributes to the toughening of foamed concrete, with increases in fiber content leading to improvements in flexural strength and peak ductility. Fu et al. [17] used the SHPB device to investigate the impact resistance of OPC mortar incorporating PPF and basalt fiber. The study revealed that an appropriate amount of PPF significantly enhances the toughness of cement-based materials. A. Kheyroddin et al. [18] used PPF and fiber-reinforced polymers (FRPs) to improve the impact behavior of concrete structures, demonstrating that the impact resistance of concrete samples can be improved through the application of PPF or glass fiber-reinforced polymer wrapping. However, most existing studies have focused on the quasi-static behavior of PPF-reinforced cementitious composites, whereas limited attention has been paid to the dynamic impact response and damage evolution of PPF-reinforced alkali-activated slag mortar. In particular, the relationship among fiber content, strain-rate sensitivity, energy absorption capacity, and internal damage morphology [19,20,21,22,23].

This study examines the effects of varying volume content on the flowability, static compressive strength, dynamic impact resistance, and dynamic crushing behavior of geopolymer mortar. It also analyzes the dynamic mechanical characteristics at different strain rates. In addition, scanning electron microscopy (SEM) and computed tomography (CT) tests were used to characterize the micromorphology and pore structure distribution of PPF bonded within the mortar. This further elucidates the toughening mechanism of PPF in geopolymer mortar.

## 2. Materials and Methods

### 2.1. Materials

The granulated blast furnace slag (GBFS) served as the binder for all mortar and paste specimens in this study [24,25]. The chemical composition and particle size distribution of GBFS are presented in Section 3.1. River sand was used as the fine aggregate in this study, with a fineness modulus of 2.36 and an apparent density of 3000 kg/m^3^. Sodium silicate (Na_2_SiO_3_) was purchased by the local supplier. The analytically pure sodium hydroxide (NaOH) purchased from local distributors is used to adjust the modulus of Na_2_SiO_3_ alkali activators. PPFs with lengths of 13 mm, diameter of 150 µm and density of 0.91 g/cm^3^ were used in this research [26].

### 2.2. Preparation and Characterization of Specimens

The water-to-binder (w/b) ratio was set at 0.55 for all mortar mixes in this study. The PPFs were added to the mortar mix at volume fractions of 0.0%, 0.2%, 0.4%, 0.6%, 0.8%, and 1.0% of the total mixture volume. A specific amount of Na_2_SiO_3_, NaOH and water was used to prepare the alkali activators, which had a modulus of 1.8 and solid content of 20%. The activators should be prepared 24 h before the experiment to ensure uniformity. After preparation, the mixture should be stirred for 300 s and then allowed to cool to room temperature for this study. The mix proportions for all mortars are presented in Table 1.

The preparation process for each group of mortar was generally consistent. First, the sand and GBFS were placed into a mechanical mixing pot and stirred for 2 min. Next, the PPFs were added to the mixing pot, and stirred for 1 min. Finally, the prepared activator solution was added to the mixing pot and stirred for another 2 min before casting the mixture into molds to produce geopolymer mortar measuring cubes with a side length of 70.7 mm and cylinders with a 74 mm diameter and 37 mm height. A vibration table is used to ensure the internal compactness of the mortar. Subsequently, the mortar, sealed in the molds, is placed in a curing room with a humidity level of no less than 95% and a temperature of (20 ± 2) °C for 24 h. For specimens with uneven surfaces, it is necessary to use a grinding machine for surface treatment before placing the specimens back in the curing room for continued curing. The mechanical performance is measured at ages of 3 days, 7 days, and 28 days.

### 2.3. Flowability of Fresh Mortars

The mortar flow test was conducted to characterize the flowability of fresh mortars for all groups under the specified vibration conditions based on Chinese Standard (GB/T 2419-2024) [27]. A caliper was used to measure the diameter of the bottom surface of the mortar in two perpendicular directions. The average value was then calculated and rounded. The larger value indicates greater flow of the fresh mortar.

### 2.4. Static Compressive Strength Test

The cubic specimens were used for static compressive strength tests. The universal testing machine (WAW-300B, Jinan, China) was used to assess the static compressive strength of mortar specimens. The static compressive strength tests were conducted in accordance with the Chinese Standard (JGJ/T 70-2009) [28]. Each group tested three samples, and the average value was recorded as the measured static compressive strength.

### 2.5. Dynamic Mechanical Properties Test

The cylinder specimens were used for dynamic mechanical properties testing. The split Hopkinson pressure bar (SHPB) device was used to investigate the dynamic mechanical properties of PPF-reinforced geopolymer mortar. Figure 1 presents a schematic diagram of the SHPB impact equipment, which primarily consists of a collection system, a compression rod system, and a loading and driving system. The fundamental principle of the SHPB experiment is based on the propagation theory of elastic stress waves in an elastic rod, relying on the one-dimensional assumption (also referred to as the plane assumption) and the uniform stress assumption. The one-dimensional assumption assumes that when stress waves propagate through an elastic rod, each cross-section of the rod consistently maintains a planar state. The uniform stress assumption indicates that the stress wave oscillates 2–3 times within the specimen, resulting in uniform stress distribution throughout the specimen [29,30].

Based on the assumption of uniform stress ($\varepsilon_{i}+\varepsilon_{r}=\varepsilon_{t}$), the three-wave method can be used to determine the average stress (${\bar{\sigma }}_{s}$), average strain (${\bar{\varepsilon }}_{s}$) and average strain rate (${\dot{\varepsilon }}_{s}$) in the specimen using Equation (1) [31]:

(1) $${\bar{\varepsilon }}_{s}\left(t\right)=\int_{0}^{t}{\dot{\varepsilon }}_{s}\left(\tau \right)d\tau$$

(2) $${\bar{\sigma }}_{s}\left(t\right)=\frac{EA}{2A_{s}}\left[\varepsilon_{i}\left(t\right)+\varepsilon_{r}\left(t\right)+\varepsilon_{t}\left(t\right)\right]$$

(3) $${\dot{\varepsilon }}_{s}\left(t\right)=\frac{C}{\delta_{S}}\left[\varepsilon_{i}\left(t\right)-\varepsilon_{r}\left(t\right)-\varepsilon_{t}\left(t\right)\right]$$

Here, *E* is the elastic modulus of the elastic rod; *C* is the stress wave velocity in the rod; *A* is the cross-sectional area of the rod; $\delta_{s}$ and *A_s_* are the cross-sectional area and the length of the mortar specimen, respectively; $\varepsilon_{i}$, $\varepsilon_{r}$ and $\varepsilon_{t}$ are the incident strain, reflected strain and transmitted strain, respectively; and t represents the duration of the stress wave.

Before conducting the experiment, the range of impact pressure was initially determined. Preliminary testing revealed that the surface of the mortar test block without PPF showed cracking at an impact pressure of 0.35 MPa. When the impact pressure increased to 0.65 MPa, the test block was completely destroyed. Therefore, the research established the impact pressure values at 0.35, 0.45, 0.55, and 0.65 MPa. The study aims to explore the variation patterns of dynamic compressive strength, ultimate toughness, and ultimate strain within a strain rate range of 50 s^−1^ to 130 s^−1^, and to analyze the strengthening mechanism of PPF on geopolymer mortar during the transition from slight cracking to partial crushing in each group of specimens [32].

### 2.6. SEM Analysis

The Zeiss GeminiSEM500 (Carl Zeiss Microscopy GmbH, Jena, Germany) was used to examine the microscopic topography of mortar after impact fracture [22]. Prior to scanning electron microscopy (SEM) testing, the sample needed to be coated with gold for 120 s to enhance its conductivity and facilitate the observation of its microscopic morphology.

### 2.7. CT Scanning Test

After dynamic mechanical properties test, the damaged cylinder specimens were used for the CT scanning test. The X-ray beams, gamma rays, and ultrasonic waves emitted by CT scanning were utilized to examine the cross-sectional surfaces of the cylinder mortar specimens. The spatial resolution of the scanned images was 50.0 μm/voxel, and the number of pixels was 1024 × 1024. The two-dimensional section was reconstructed in three dimensions to further characterize the internal void structure change of geopolymer mortar after impact [33,34,35].

## 3. Results and Discussions

### 3.1. Characterization of Raw Materials

Its oxide compositions, based on X-ray fluorescence (XRF) analysis, are presented in Table 2. The particle size distributions of GBFS were determined using a laser diffraction technique with a Mastersizer 2000 (Malvern Instruments Ltd., Malvern, UK), as shown in Figure 2. The median particle size (d50) of GBFS is 13.18 µm.

### 3.2. Flowability

The flowability results of the geopolymer mortar with varying PPF content are presented in Figure 3. The flowability of the plain mortar (Ctrl) is measured at 253 mm. The flowability of the geopolymer mortar with PPF volume content of 0.2% is 248 mm. As the PPF content increases, the flowability gradually decreases [36]. The mortar with 1.0% volume content exhibits the flowability of 224 mm, representing the reduction of 11.46% when compared with the Ctrl sample. The primary reasons for this decrease are as follows: (1) A small amount of PPF has good dispersion within the geopolymer mortar, forming an effective three-dimensional skeletal structure that restricts the flow of the mortar. (2) The irregular distribution of PPF within the mortar matrix creates a network of varying spatial structures. As the dosage increases, the net content of PPF per unit volume also rises, resulting in a greater division of the mortar into smaller spaces. This increased division leads to higher friction resistance within the mortar, which can inhibit the flow of the geopolymer mortar.

### 3.3. Static Compressive Strength

Figure 4 shows the static compressive strength of geopolymer mortar at 3, 7, and 28 days with varying PPF content. In the present experiments, for each mixture and curing age, three specimens were tested, and the plotted values are arithmetic means. The compressive strength at ages exhibits a consistent trend. At a PPF volume content of 0.2%, the incorporation of a small amount of PPF enhances the static compressive strength of geopolymer mortar. However, when the PPF content exceeds 0.2%, a slight decrease in compressive strength is observed. This reduction is primarily attributed to the fact that a limited amount of PPF fills the pores in the mortar. Additionally, the random distribution of PPF creates a skeletal effect, which effectively impedes the formation and propagation of microcracks. Conversely, the excessive amount of PPF may clump together during mixing, resulting in uneven distribution and a subsequent reduction in compressive strength [37].

### 3.4. Failure Morphology of Geopolymer Mortar

The typical failure patterns of geopolymer mortars with varying PPF content under different levels of impact air pressure are shown in Figure 5. It is evident that mortars with different PPF content primarily exhibit axial splitting tensile failure. As the air pressure increases, the degree of mortar crushing intensifies, resulting in a greater number of fragments and a gradual reduction in the size of the crushed particles. The crushing particle size of PPF-reinforced geopolymer mortar has larger crushing particle size compared with the plain mortar (Ctrl group). Micro-cracks appear on the surface at an impact air pressure of 0.35 MPa, while the surface of the specimen remains flat with no debris falling off. In contrast, ordinary mortar exhibits large cracks, indicating that PPF significantly enhances the reinforcement of mortar under low air pressure. As the air pressure increases to 0.45 MPa, 0.55 MPa, and 0.65 MPa, ordinary mortar is completely crushed, resulting in progressively smaller particle sizes. However, PPF-reinforced geopolymer mortar only displays increased surface cracks without any penetrating cracks.

Different PPF contents in mortar exhibit varying forms of damage under the same impact air pressure. At a low air pressure of 0.35 MPa, the surface micro-cracks in the specimens gradually decreased as the PPF content increased. However, when the impact air pressure reached 0.45 MPa, some specimens displayed an increase in both the number and width of surface cracks, and fragments of the composite PPF mortar began to detach. The mortar specimens with higher PPF content primarily showed an increase in micro-cracks without the formation of large cracks. As the impact air pressure continued to rise to 0.55 MPa and 0.65 MPa, the mortars with low PPF contents of 0.2% and 0.4% by volume developed penetrating cracks, and the degree of crushing intensified. Conversely, when the PPF content reached 0.8% and 1.0%, the surface cracks were reduced, but the mortar did not separate. Based on the failure patterns observed in the present study, the improvement at higher PPF contents is attributed to fiber bridging, crack deflection, and fiber pull-out. This interpretation is consistent with existing studies on fiber-reinforced geopolymer and cementitious materials [38,39,40].

The observed phenomenon primarily results from the irregular distribution of PPF within geopolymer mortar. This distribution facilitates the formation of bridging and reinforcement within the mortar, resulting in enhancing overall integrity. The PPF exhibits excellent tensile strength, enabling it to withstand damage induced by dynamic loads. As the impact load increases, the PPF within the interface transition zone (ITZ) of the mortar may undergo deformation and, in some cases, may become dislodged or pulled out. Consequently, when subjected to impact loads, PPF can absorb a portion of the energy and alter the internal stress transfer within the mortar. This process alleviates the stress concentration phenomenon at the crack tip within geopolymer mortar, further inhibiting the initiation and propagation of cracks within the material.

### 3.5. Dynamic Compressive Strength

The results of the SHPB tests on PPF-reinforced geopolymer mortar are presented in Table 3. It can be seen that the dynamic stress–strain curve exhibits a brief linear phase in the initial stage. This observation indicates that during the impact process, once the mortar is subjected to the impact load, the mortar specimen rapidly transitions into the elastic deformation stage. This behavior is primarily attributed to the rapid closure of the internal pores within the mortar specimen, which occurs as it approaches the compaction stage [41]. At this point, the energy from the impact rod is converted into the elastic potential energy of the specimen. When the elastic limit stress is reached, the specimen enters the crack expansion stage [42], where a significant portion of the energy is utilized to create new cracks and induce crack damage. The stress progresses into the plastic deformation stage as the strain increases. The energy stored within the mortar is dissipated due to the failure of its internal structure and ultimately reaches the yield stress. In the later stages, the impact-induced cracks continue to expand and propagate, leading to the penetration of the cracks through the specimen and resulting in failure. Consequently, the stress decreases rapidly with only a slight increase in strain.

Table 3 presents the mechanical properties of geopolymer mortar. It can be observed from Table 3 that the dynamic compressive strength of plain geopolymer mortar and PPF-reinforced geopolymer mortar increases with the strain rate. This increase is primarily attributed to the rise in impact air pressure and the kinetic energy carried by the SHPB impact rod. Upon contact between the SHPB impact rod and the specimen, the specimen undergoes compression, initiating energy transfer. Previous studies have indicated that the energy required for crack initiation is significantly greater than that needed for crack propagation [29]. Consequently, as the strain rate increases, the failure mode of the impact-loaded specimen is predominantly characterized by an increase in the number of cracks, and the energy transfer process occurs more rapidly. This rapid transfer prevents the PPF-reinforced geopolymer mortar from accumulating energy instantaneously, resulting in a brief buffer stage before the specimen undergoes impact deformation. As a result, the specimen can only resist the energy and impulse from the impact by increasing its own stress. At the same time, it can be seen that the impact toughness and peak stress of plain geopolymer mortar are relatively low. This is primarily due to the inability of the energy transfer to be fully realized when the plain geopolymer mortar specimen is subjected to impact loading, resulting in diminished impact toughness. The impact toughness of PPF-reinforced geopolymer mortar increased by 99.3% relative to plain geopolymer mortar. This enhancement is mainly attributed to fiber bridging at microcracks: under impact loading, the PPF carries additional stress, while compressive deformation of the fine aggregate increases the frictional resistance and adhesion at the aggregate-fiber interface. Together, these effects inhibit the propagation of some cracks, improve the specimen’s deformation capacity, and prevent rapid crack growth.

### 3.6. Peak Stress and Strain

Figure 6 shows the fitted relationship between dynamic compressive strength and average strain rate. The corresponding linear relationships are represented by Equations (4)–(9):

(4) $$f_{\mathrm{PPF}-0.0\%}=0.333\bar{\dot{\varepsilon }}+9.145$$

(5) $$f_{\mathrm{PPF}-0.2\%}=0.415\bar{\dot{\varepsilon }}+5.315$$

(6) $$f_{\mathrm{PPF}-0.4\%}=0.288\bar{\dot{\varepsilon }}+16.672$$

(7) $$f_{\mathrm{PPF}-0.6\%}=0.272\bar{\dot{\varepsilon }}+22.471$$

(8) $$f_{\mathrm{PPF}-0.8\%}=0.256\bar{\dot{\varepsilon }}+26.688$$

(9) $$f_{\mathrm{PPF}-1.0\%}=0.299\bar{\dot{\varepsilon }}+27.759$$

These results indicate that within the strain rate range of 50 s^−1^ to 130 s^−1^, the dynamic compressive strength of six different PPF contents in mortar increases as the average strain rate rises. This phenomenon is primarily due to the transformation of the internal mechanical response of the specimen from a one-dimensional stress state to a one-dimensional strain state during the impact loading process, while lateral strain remains constrained [43]. This limiting effect is positively correlated with changes in strain rate. This causes the specimen to approximate a confining pressure state, which results in strain rate hardening during the impact process.

At the same strain rate, the dynamic compressive strength increased continuously with PPF content, and the mortar containing 1.0% PPF achieved the highest value. This is mainly because the addition of fibers can effectively mitigate the drying shrinkage of geopolymer mortar [44]. When the specimen is subjected to impact damage, PPF also undergoes deformation, transferring some energy during this process. In contrast to plain geopolymer mortar, it does not directly experience brittle failure. The intricate distribution of PPF within the mortar matrix creates a bridging effect, leading to the enhancement on dynamic compressive strength. Compared with the control group, the peak stress increased by 54.5%, 39.8%, and 29.6% at strain rates of 60 s^−1^, 90 s^−1^, and 120 s^−1^, respectively. It is evident that the increase in peak stress at lower average strain rates is greater than that at higher strain rates, indicating that PPF-reinforced geopolymer mortar exhibits superior damage resistance at low strain rates when compared with high strain rates. At low strain rates, PPF effectively suppresses crack propagation and alleviates the stress concentration associated with cracks in the specimen. Conversely, at high strain rates, geopolymer mortar bears greater impact loads, and the bridging effect formed at the interface between PPF and the mortar is partially compromised, diminishing its ability to suppress cracks. When the specimen is subjected to impact loads, the energy conversion efficiency decreases, leading to a reduction in the dynamic compressive strength of PPF-reinforced geopolymer mortar.

### 3.7. Dynamic Growth Factors

The ratio of dynamic compressive strength to static compressive strength is defined as the dynamic growth factor (DIF), which is used to evaluate the influence of strain rate on dynamic compressive strength [42,45]. As illustrated in Figure 7, it can be observed that under high strain rate dynamic impact, the DIF for all six types of mortar increases with the average strain rate. The geopolymer mortar mixed with PPF exhibits a higher DIF when compared with the control group, indicating that PPF can improve the strain rate sensitivity of geopolymer mortar. Therefore, the dynamic strength of geopolymer mortar continues to improve, achieving a maximum DIF of approximately 2.1. In the analysis of the static compressive strength of PPF-reinforced geopolymer mortar, it was observed that PPF has a minimal effect on the static compressive strength of PPF-reinforced geopolymer mortar. A small addition of PPF can improve the compressive strength of PPF-reinforced geopolymer mortar, with the 0.2% volume addition of PPF resulting in good static compressive strength, which is essentially comparable to that of plain geopolymer mortar. However, the DIF at high strain rates can reach 1.4. Although the static compressive strength of geopolymer mortar with high PPF content slightly decreased, with a maximum reduction of 7.68% compared with the control group, the highest DIF can reach 2.129. Although PPF has a minor impact on static compressive strength, it significantly contributes to dynamic impact resistance due to its bridging effect and the inherent toughness of PPF.

### 3.8. Ultimate Toughness

Ultimate toughness characterizes the ability of materials to absorb energy from loading until failure, serving as a comprehensive reflection of both material strength and ductility [46]. The ultimate toughness of PPF-reinforced geopolymer mortar shows a linear relationship with strain rate. By combining quasi-static compressive strength and dynamic compressive strength, it can be seen that geopolymer mortar exhibits improved performance as the PPF content increases. To further investigate the trend of ultimate toughness in PPF mortar with varying dosages under different strain rates, curve fitting was conducted within the range of 50 s^−1^ to 130 s^−1^ and compared with plain mortar, as shown in Figure 8.

It can be seen that the average strain rate ranges from 50 s^−1^ to 130 s^−1^, and the ultimate toughness of both plain mortar and PPF-reinforced geopolymer mortar increases with the average strain rate. This indicates that the ultimate toughness of both types of mortar exhibits a significant strain rate effect. At the same strain rate, the ultimate toughness of PPF-reinforced geopolymer mortar is greater than that of plain mortar, suggesting that mortar mixed with PPF has higher energy transfer efficiency than plain mortar. When mortar is subjected to impact load, the primary energy consumption arises from the formation of initial cracks and the generation of new cracks, with the latter being the predominant factor [29,47]. The bridging effect created by PPF in the mortar effectively mitigates the formation of microcracks. Additionally, PPF possesses a certain degree of toughness. Upon experiencing impact loads, PPF-reinforced geopolymer mortar enters a compaction phase, during which the frictional resistance between the internal PPF fibers and the mortar increases. During the processes of mortar fragmentation and detachment, the deformation resulting from fiber elongation also contributes to energy consumption.

### 3.9. SEM Analysis

SEM was used to observe the mortar microstructure of PPF-reinforced geopolymer mortar cured for 28 days, as shown in Figure 9. Figure 9a clearly reveals the presence of cracks of varying scales within the geopolymer mortar without PPF, which adversely affects its mechanical properties. In contrast, Figure 9b shows that the internal microcracks in the geopolymer mortar containing PPF are significantly reduced. This reduction is primarily attributed to the random distribution of PPF within the mortar, which inhibits crack development and consequently diminishes crack scale. It can be observed from Figure 9c that PPF can effectively penetrate the matrix, which can improve the interfacial bonding strength to a certain extent [33,48]. When the mortar is damaged, some fibers may become deformed or even pulled out due to tensile forces, as clearly seen in Figure 9d. It can also be seen that the fiber surfaces remain relatively intact and that the geopolymer matrix is not corroded, indicating that PPF has good durability and stability. Furthermore, Figure 9e shows scratches on the surfaces of some fibers, which are mainly caused by the load of the mortar during the fiber extraction process. This process consumes external energy, which mitigates the stress concentration phenomenon within the matrix, thereby inhibiting crack propagation and matrix cracking, and improving the ductility of the matrix. It should be noted that pre-impact SEM analysis of companion specimens was not included in the original experimental design; therefore, the SEM results are not used to establish a before-and-after damage-evolution comparison.

### 3.10. CT Analysis

CT scanning was performed on representative specimens with PPF volume contents of 0.6%, 0.8%, and 1.0% after they were subjected to an impact air pressure of 0.55 MPa. The internal voids were characterized by their equivalent diameter and volume [34,35]; Figure 10 presents the number of detected voids as a function of equivalent diameter (0–4 mm) on a logarithmic scale, and Figure 11 presents the volume of each detected void against its equivalent radius (0–3 mm).

For all three specimens, the number of internal voids decreased sharply with increasing diameter: voids smaller than 0.5 mm dominated the void population, whereas voids larger than 2 mm were rare. In the PPF-0.6% specimen, about 10^4^ voids were detected at an equivalent diameter of about 0.1 mm, and the counts decreased to about 180, 30, 10, and 5 at diameters of 0.5, 1.0, 1.5, and 2.0 mm, respectively. The PPF-0.8% specimen contained a comparable number of very small voids but fewer voids in the intermediate range, with about 145 and 17 voids at 0.5 and 1.0 mm, respectively. The PPF-1.0% specimen showed the fewest small voids, with about 10^3^ voids at 0.1 mm and 95 voids at 0.5 mm, while its counts at 1.5–2.0 mm were similar to those of the PPF-0.8% specimen.

The volume–radius distributions further revealed the difference in internal damage [47,48]. In the PPF-0.6% specimen, about 31 voids with a volume larger than 4 mm^3^ were identified, mostly at equivalent radii of 0.2–1.1 mm, and the maximum void volume reached about 12.8 mm^3^ at a radius of about 1.0 mm. In the PPF-0.8% specimen, only two voids exceeded 4 mm^3^, with the largest volume of about 5.1 mm^3^ at a radius of about 1.8 mm. In the PPF-1.0% specimen, five voids exceeded 4 mm^3^, with the largest volume of about 8.3 mm^3^ at a radius of about 2.3 mm and the remaining large voids located at radii of 1.9–2.9 mm. These results show that increasing the PPF content substantially reduced both the number and the volume of large internal voids, although the few remaining large voids in the PPF-1.0% specimen tended to appear at larger radii.

The CT observations are consistent with the failure morphology and dynamic toughness results. The abundant large voids in the PPF-0.6% specimen indicate the most severe internal damage, whereas the suppression of large-void formation in the PPF-0.8% and PPF-1.0% specimens indicates that PPF inhibited the initiation and propagation of internal cracks. The CT results therefore provide mesoscopic evidence for the toughening mechanism of PPF in geopolymer mortar under impact loading.

## 4. Discussion

This study shows that PPF reinforcement is an effective way to improve the dynamic impact resistance of alkali-activated slag geopolymer mortar without significantly compromising its static properties. Flowability decreased with increasing PPF content, whereas the static compressive strength changed little: 0.2% PPF gave a slight improvement, and higher contents reduced it by at most 7.68%. The non-monotonic change in static compressive strength reflects two competing mechanisms: pore filling and crack bridging by a small amount of PPF, versus fiber agglomeration and weak fiber–matrix interfaces at higher contents. In contrast, the dynamic response was markedly enhanced; the peak stress, DIF, and ultimate toughness increased with strain rate and fiber content, and the impact toughness increased by 99.3% relative to the plain mortar.

The strain-rate sensitivity is consistent with the general behavior of cementitious materials: lateral inertia constrains deformation at high strain rates, transforming the stress state to approximately triaxial and producing strain-rate hardening [20,21,42,43]. PPF strengthens this response by sharing tensile stress across crack planes and increasing the energy required for crack initiation and propagation. The peak-stress increases of 54.5%, 39.8%, and 29.6% at about 60, 90, and 120 s^−1^, respectively, indicate that PPF is more effective at lower strain rates, where bridging remains efficient; at higher rates, rapid crack growth may exceed the fiber–matrix bond capacity.

The CT results provide a quantitative mesoscopic explanation. The PPF-0.6% specimen contained about 31 voids larger than 4 mm^3^, with a maximum volume of about 12.8 mm^3^, whereas the PPF-0.8% and PPF-1.0% specimens contained only about 2 and 5 such voids, respectively. PPF therefore mainly suppressed the formation of intermediate- and large-sized voids. Notably, the PPF-1.0% specimen did not have the fewest large voids; its remaining large voids were larger in radius (1.9–2.9 mm). Its excellent dynamic performance thus cannot be attributed to absolute void volume alone; fiber bridging, crack arrest, and energy dissipation are likely the dominant factors, which also explains the slight static-strength reduction but marked toughness increase at high PPF contents.

These findings extend the established benefits of PPF in Portland cement-based composites to alkali-activated slag mortar, and the combined SHPB, SEM, and CT evidence links macroscopic dynamic properties to internal damage evolution. Limitations include a single water-to-binder ratio of 0.55, one fiber geometry, PPF contents of 0–1.0%, strain rates of about 50 s^−1^–130 s^−1^, and CT scanning only at 0.55 MPa. The quantitative relationship between fiber content and crack connectivity or porosity remains to be established, and high PPF contents reduce flowability, which may cause uneven fiber distribution in practice. Future work should quantify crack connectivity from CT images, explore fiber geometry and hybrid fibers over wider strain rates, and develop mesoscale damage models.

The SWOT results do not support a single optimum PPF content for all applications, are presented in Table 4. A content of 0.2% is preferable when workability and static strength are the primary requirements. A content of 0.8–1.0% is more appropriate when dynamic impact resistance and energy absorption are prioritized. The 0.4–0.6% range should be selected only when a specific balance between workability and dynamic performance is required and can be verified through dispersion and batch-consistency testing.

The SWOT assessment is bounded by the tested water-to-binder ratio, single fiber geometry, PPF content range, strain-rate range, and CT condition. Economic, durability, and industrial factors are included as opportunities and threats but were not experimentally quantified in this study.

**Table 4:** Dosage-specific SWOT analysis of the engineering performance of PPF-reinforced alkali-activated slag geopolymer mortar.

PPF Content	Strengths	Weaknesses	Opportunities	Threats
Low dosage 0.2%	Best flowability; static strength maintained.	Limited dynamic gain.	Repair and precast applications.	Impact performance near control.
Intermediate dosage 0.4–0.6%	Improved dynamic performance.	Lower flowability; 0.6%: most large voids.	Moderate-impact formulations.	Clustering and batch variability.
High dosage 0.8–1.0%	Highest DIF and toughness; shape retained under high pressure.	1.0%: 224 mm flowability; static strength down by up to 7.68%.	Impact-resistant elements; recycled-PPF pilots.	Cost, durability, scale-up, standards.

Environmental, economic, and social dimensions should be considered together when assessing the proposed PPF-reinforced alkali-activated slag mortar. The mortar may reduce OPC demand, valorize GBFS, and extend service life through improved impact resistance, but a full life-cycle assessment is needed to include activator production, transport, energy use, and end-of-life [49]. Economic benefits depend on reduced repair and downtime relative to local material costs, while social benefits require appropriate standards, workforce capability, and acceptance by producers, contractors, and users. Horizontal communication among material scientists, engineers, analysts, suppliers, producers, contractors, testing organizations, and regulators can align technical performance with sustainability criteria. Vertical communication from laboratory development through design, construction, operation, and policy can support staged implementation. Both are needed to translate these material-level findings into practice, and neither was assessed in this study [50].

## 5. Conclusions

(1) The flowability of fresh geopolymer mortar decreased monotonically with increasing PPF content, reaching 224 mm at a volume content of 1.0% (11.46% lower than the plain mortar), whereas the static compressive strength was only slightly affected: a small addition of 0.2% produced a slight improvement, and higher contents caused a maximum reduction of 7.68%.

(2) The dynamic compressive strength, DIF, and ultimate toughness of all mortars increased with increasing strain rate, and PPF enhanced the strain-rate sensitivity of geopolymer mortar. The mortar with 1.0% PPF exhibited the best dynamic performance, with a maximum DIF of 2.129, an increase in impact toughness of 99.3% relative to the plain mortar, and peak-stress increases of 54.5%, 39.8%, and 29.6% at strain rates of about 60 s^−1^, 90 s^−1^, and 120 s^−1^, respectively.

(3) CT analysis showed that increasing the PPF content substantially reduced both the number and the volume of large internal voids: about 31 voids larger than 4 mm^3^ were detected in the PPF-0.6% specimen (maximum about 12.8 mm^3^), whereas only about two and five such voids were found in the PPF-0.8% and PPF-1.0% specimens, respectively. Combined with the failure-morphology and SEM observations of fiber bridging and pull-out, this indicates that PPF improves the impact resistance of geopolymer mortar mainly by suppressing internal crack formation and propagation. Within the investigated range (w/b of 0.55, PPF content of 0–1.0%, strain rates of 50 s^−1^–130 s^−1^), a PPF content of 1.0% is recommended for impact-resistant applications; quantitative CT analysis of crack connectivity over a wider parameter range is needed to further generalize these conclusions.

Overall, PPF-reinforced alkali-activated slag mortar offers a promising material-level contribution to sustainable impact-resistant construction. Its environmental, economic, and social benefits, together with the communication pathways required for scale-up, should be verified through integrated assessment and pilot-scale stakeholder validation.

## Figures and Tables

**Figure 1:**
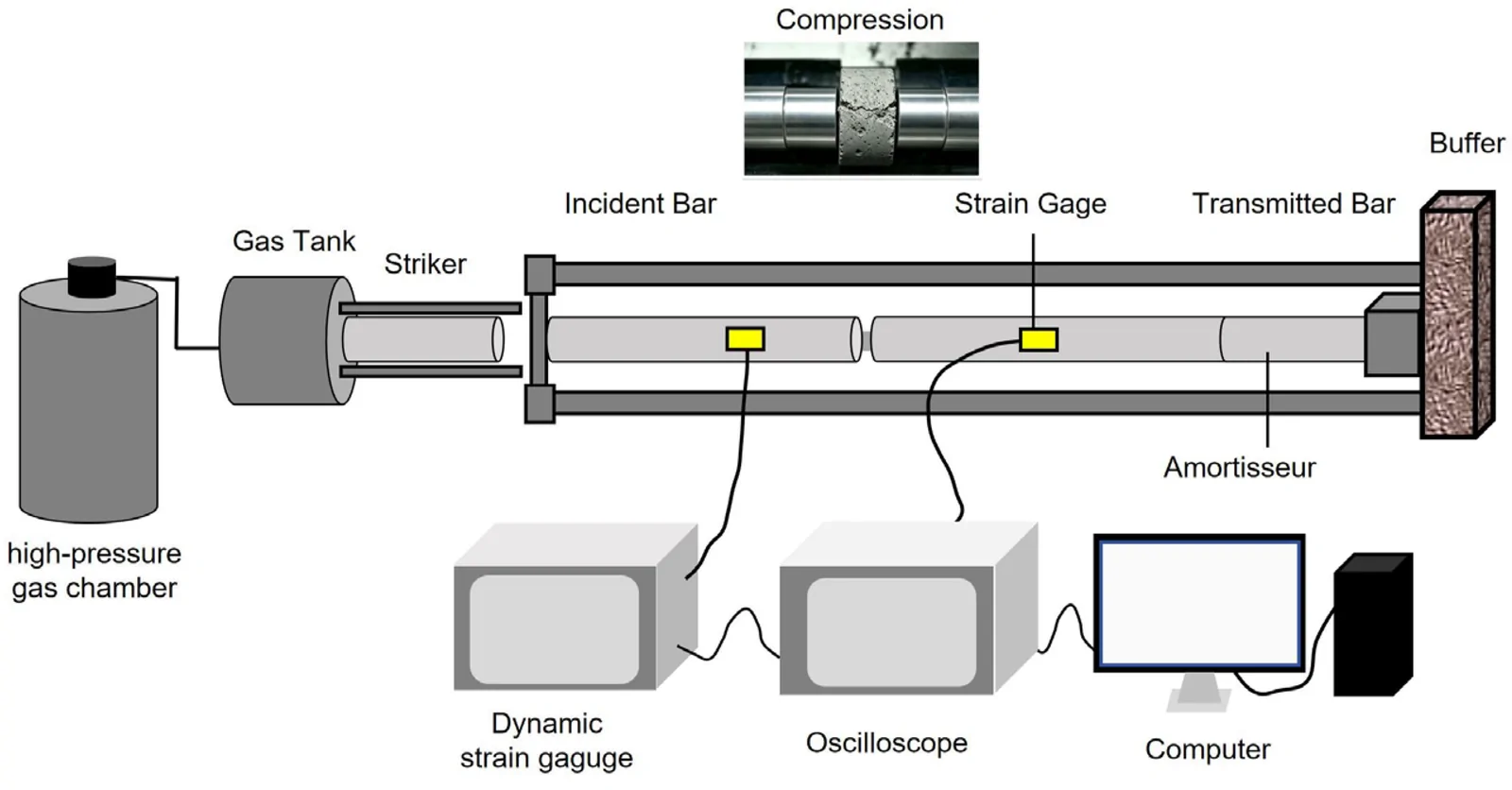
Schematic diagram of the SHPB set.

**Figure 2:**
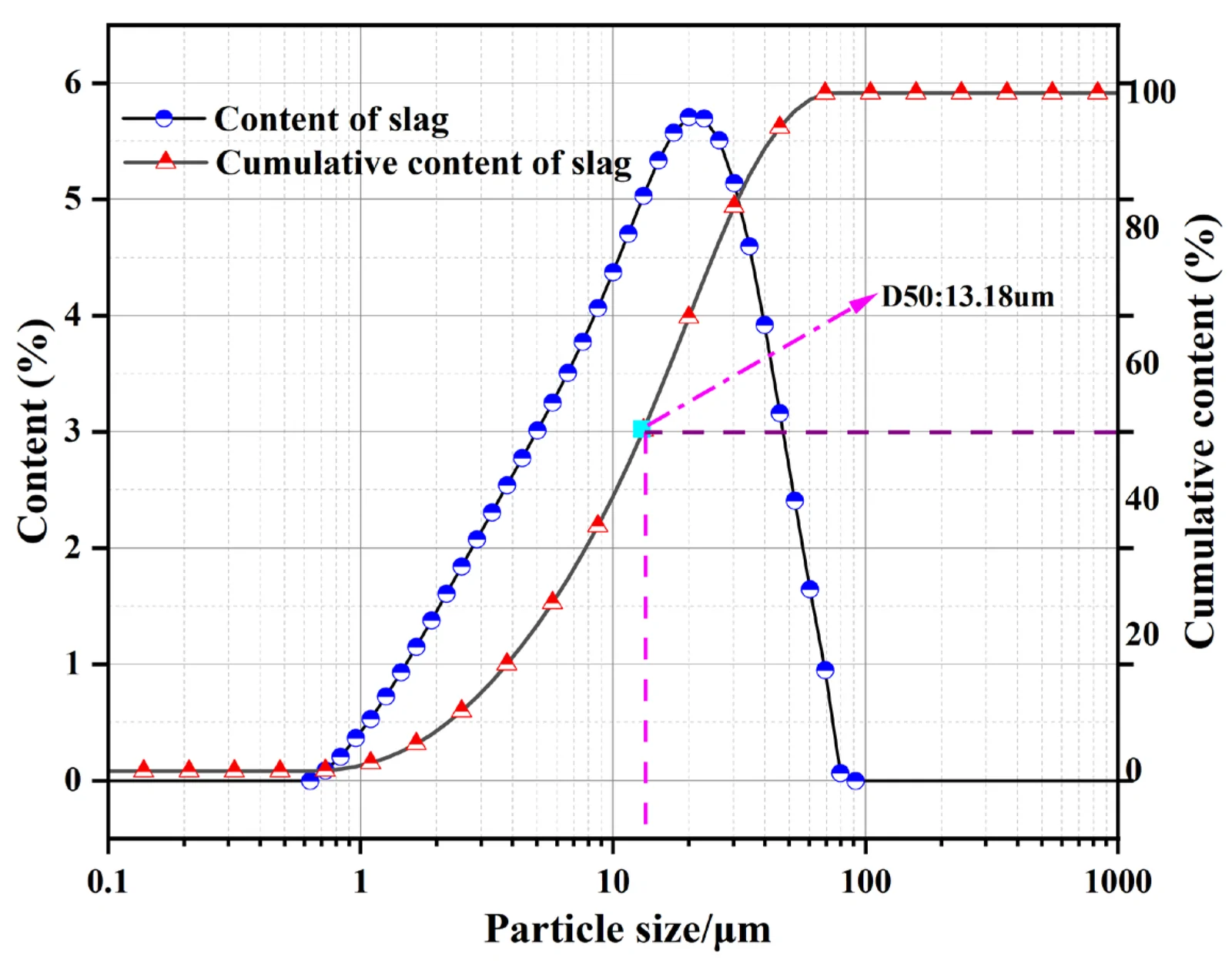
Particle size distribution of GBFS.

**Figure 3:**
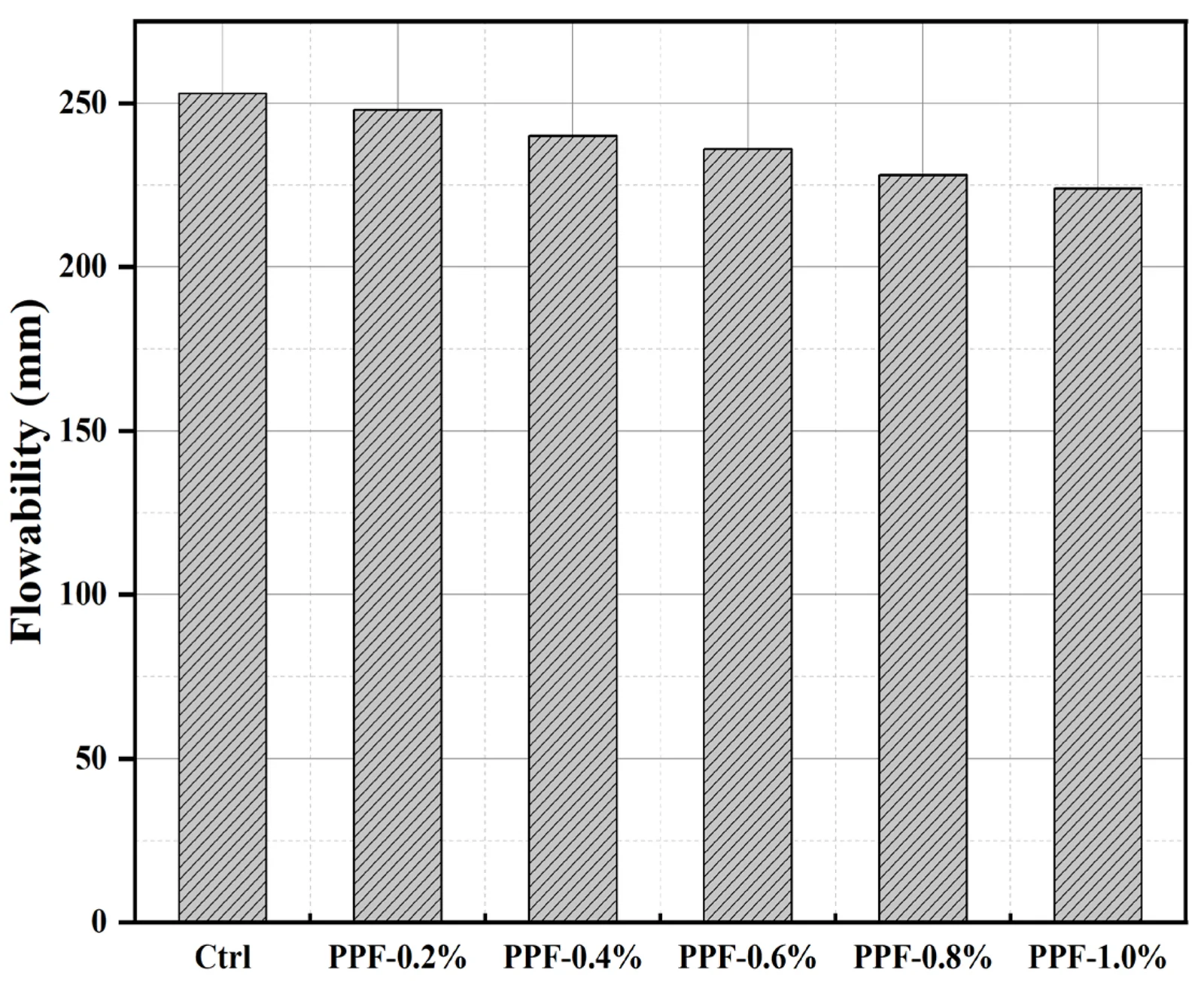
Flowability of geopolymer mortar.

**Figure 4:**
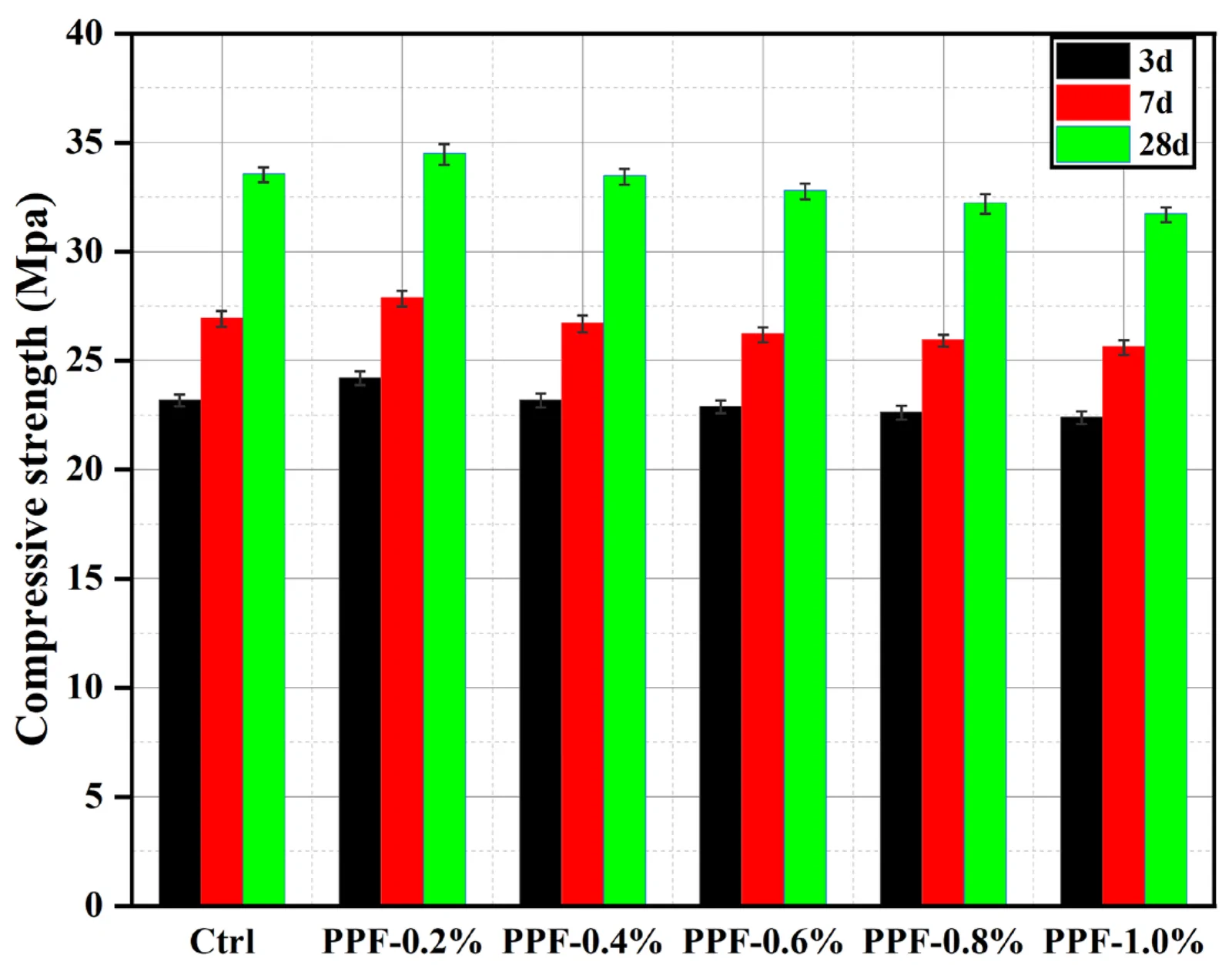
Compressive strength of geopolymer mortar at different ages with different PPF content.

**Figure 5:**
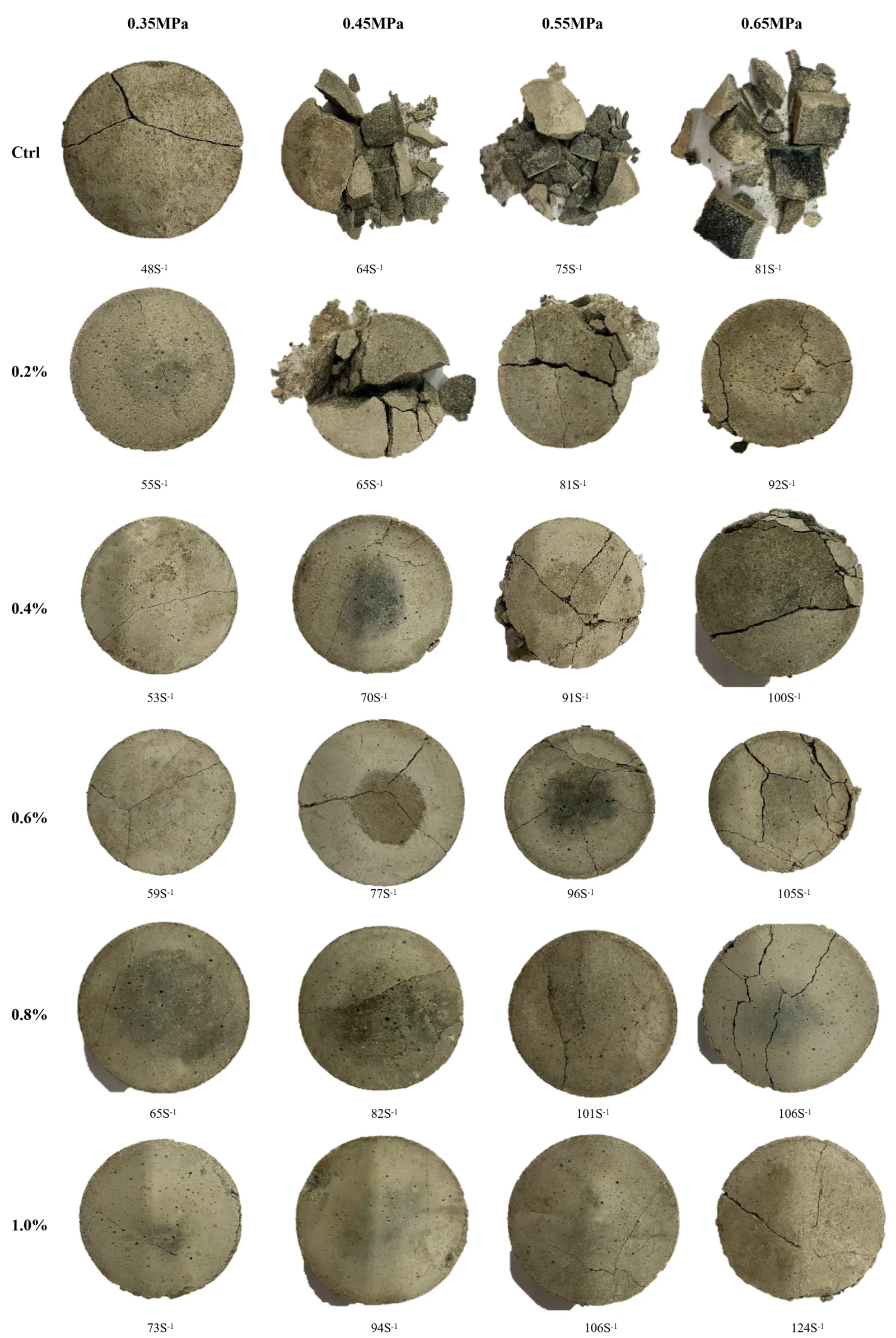
Crushing forms of geopolymer mortar with different PPF content under different impact loads.

**Figure 6:**
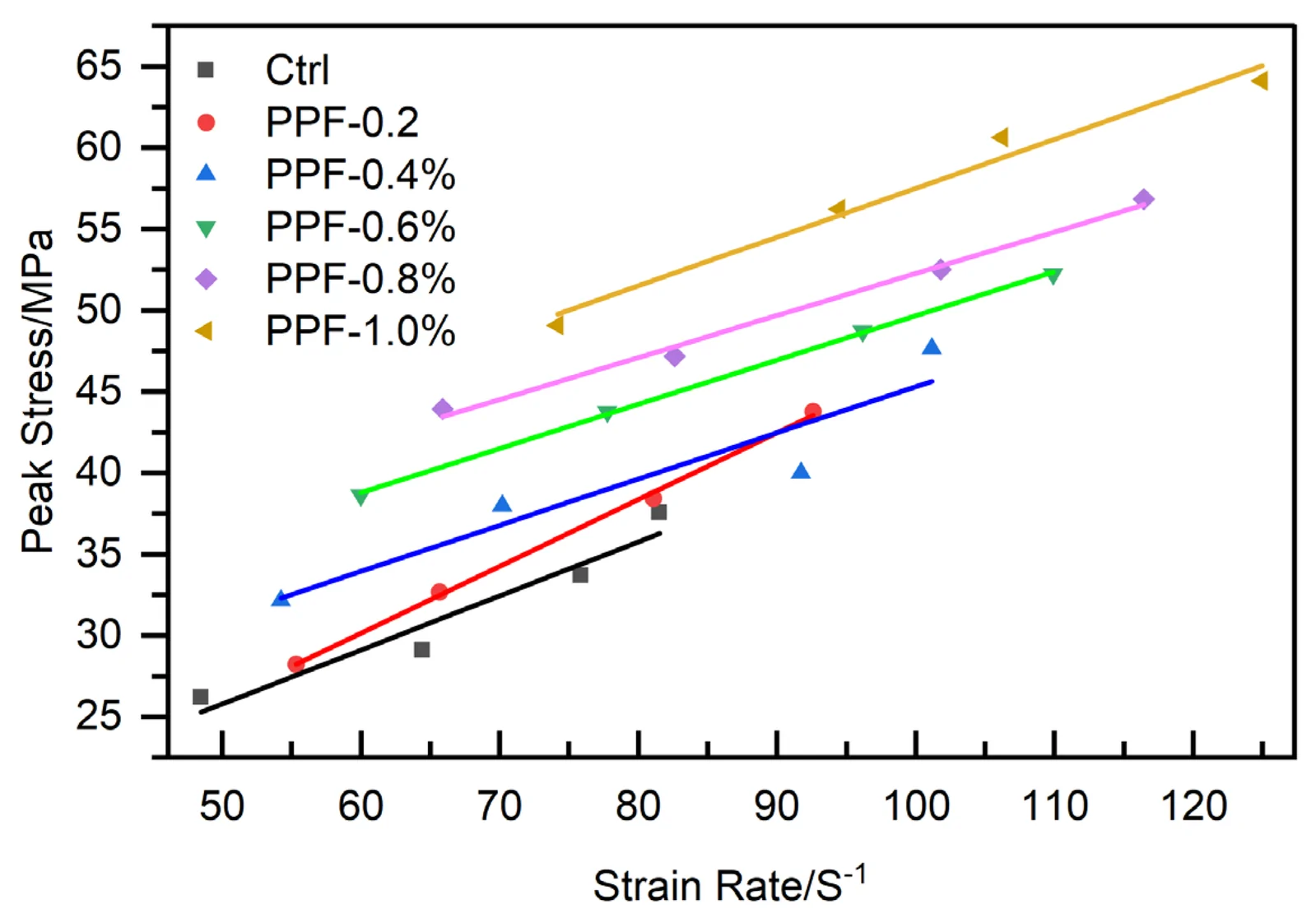
Relationship fitting curves of the dynamic compressive strength versus the average strain rates of the mortar specimens.

**Figure 7:**
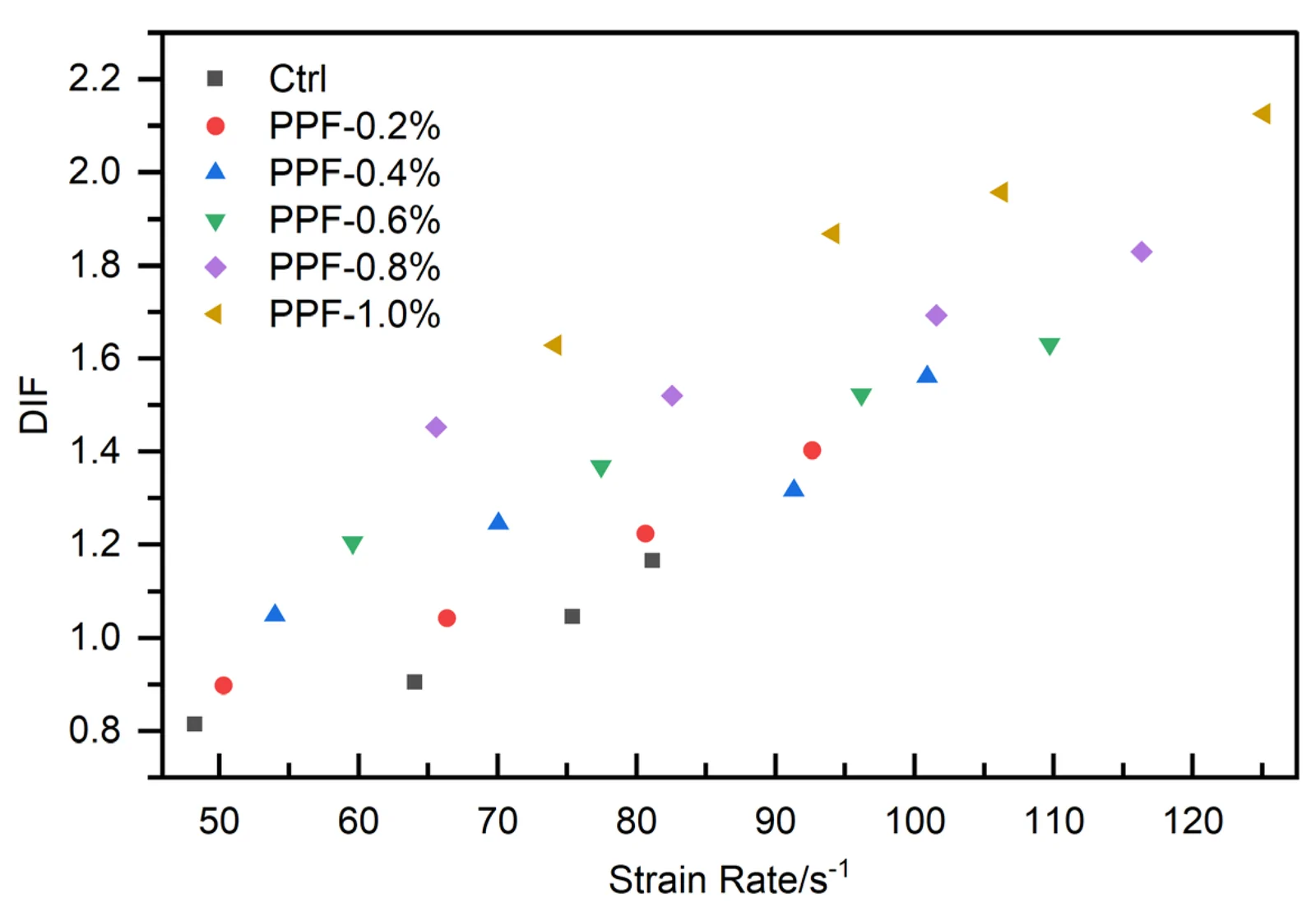
DIF of the mortar specimens.

**Figure 8:**
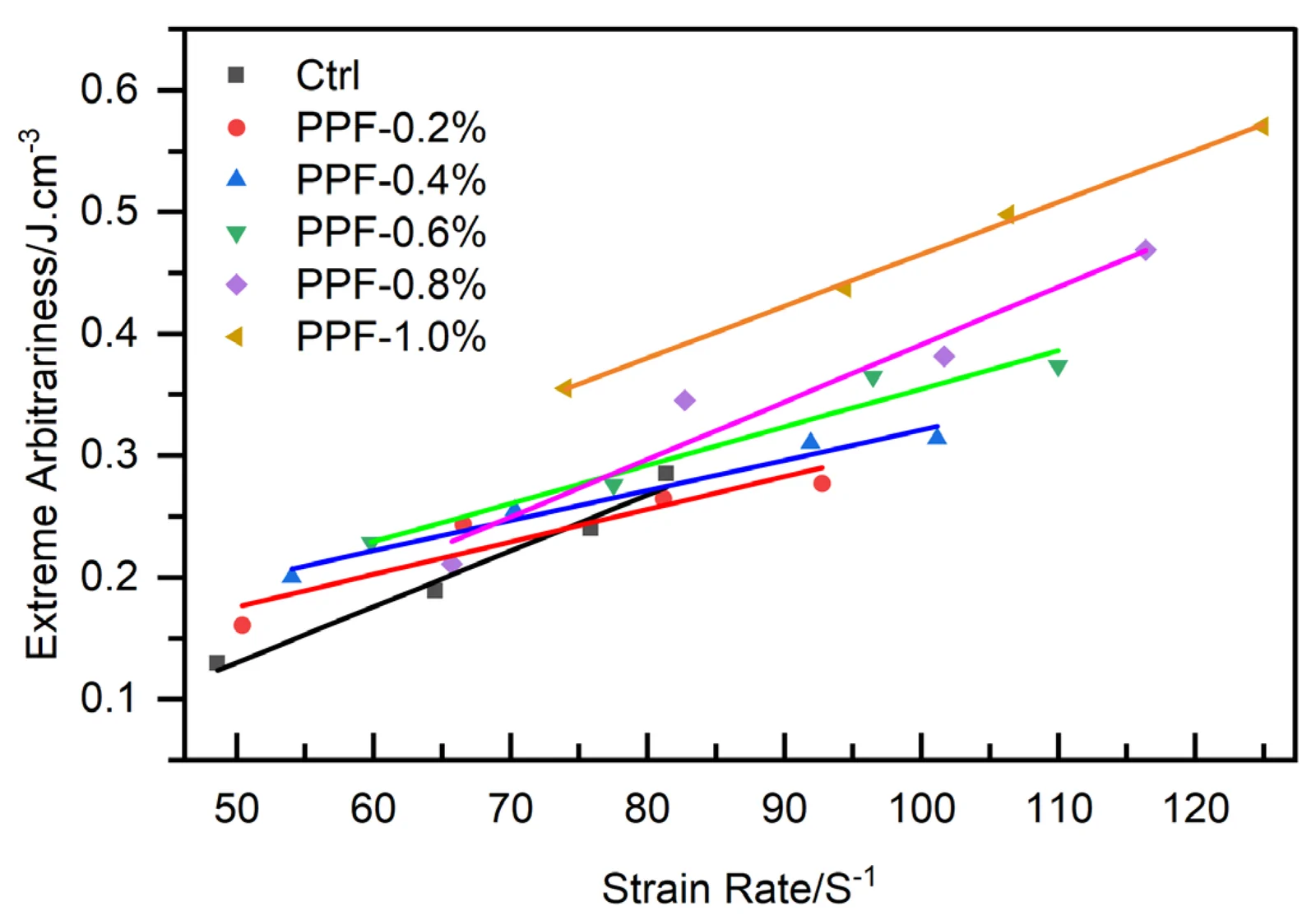
Relationship fitting curves of the dynamic ultimate toughness versus the average strain rates of the mortar specimens.

**Figure 9:**
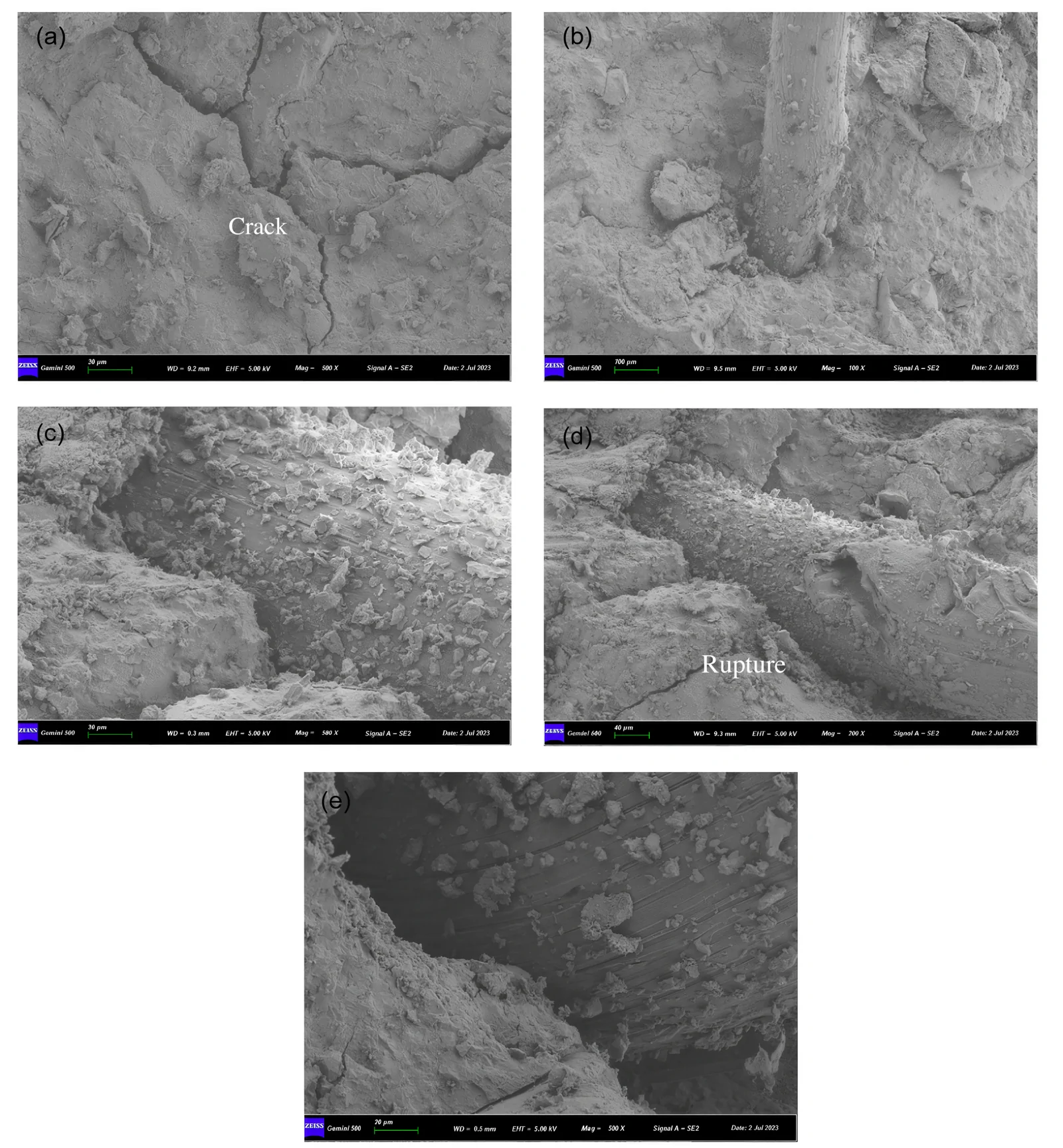
Microstructure of geopolymer mortar (**a**) Cracks in mortar without PPF. (**b**) Reduced microcracks after PPF reinforcement. (**c**) PPF penetrating the matrix and improving interfacial bonding. (**d**) Deformed/pulled-out PPF with intact surfaces and an uncorroded matrix. (**e**) Fiber-surface scratches indicating energy dissipation during pull-out.

**Figure 10:**
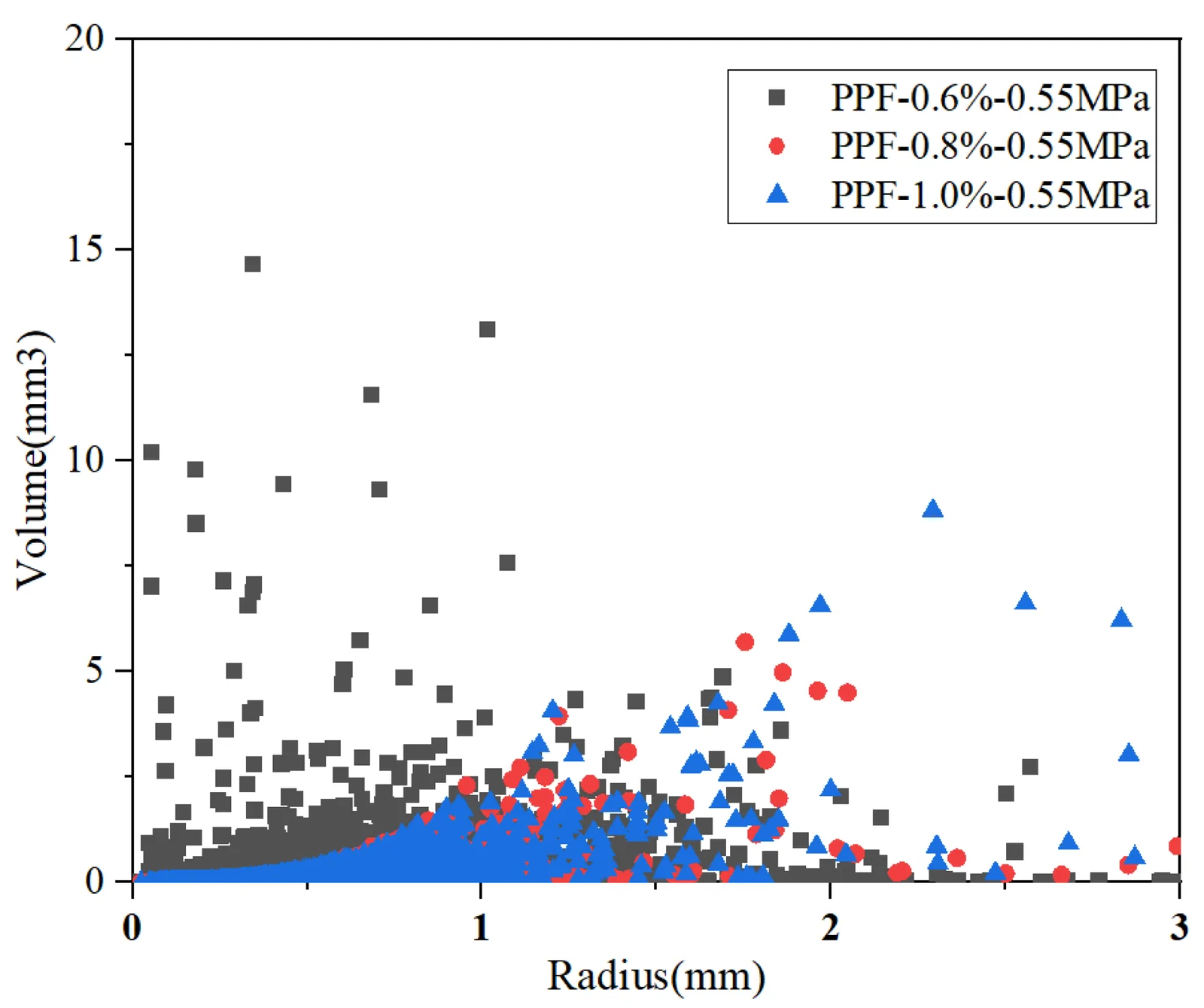
Pore-fracture size distribution from CT scanning.

**Figure 11:**
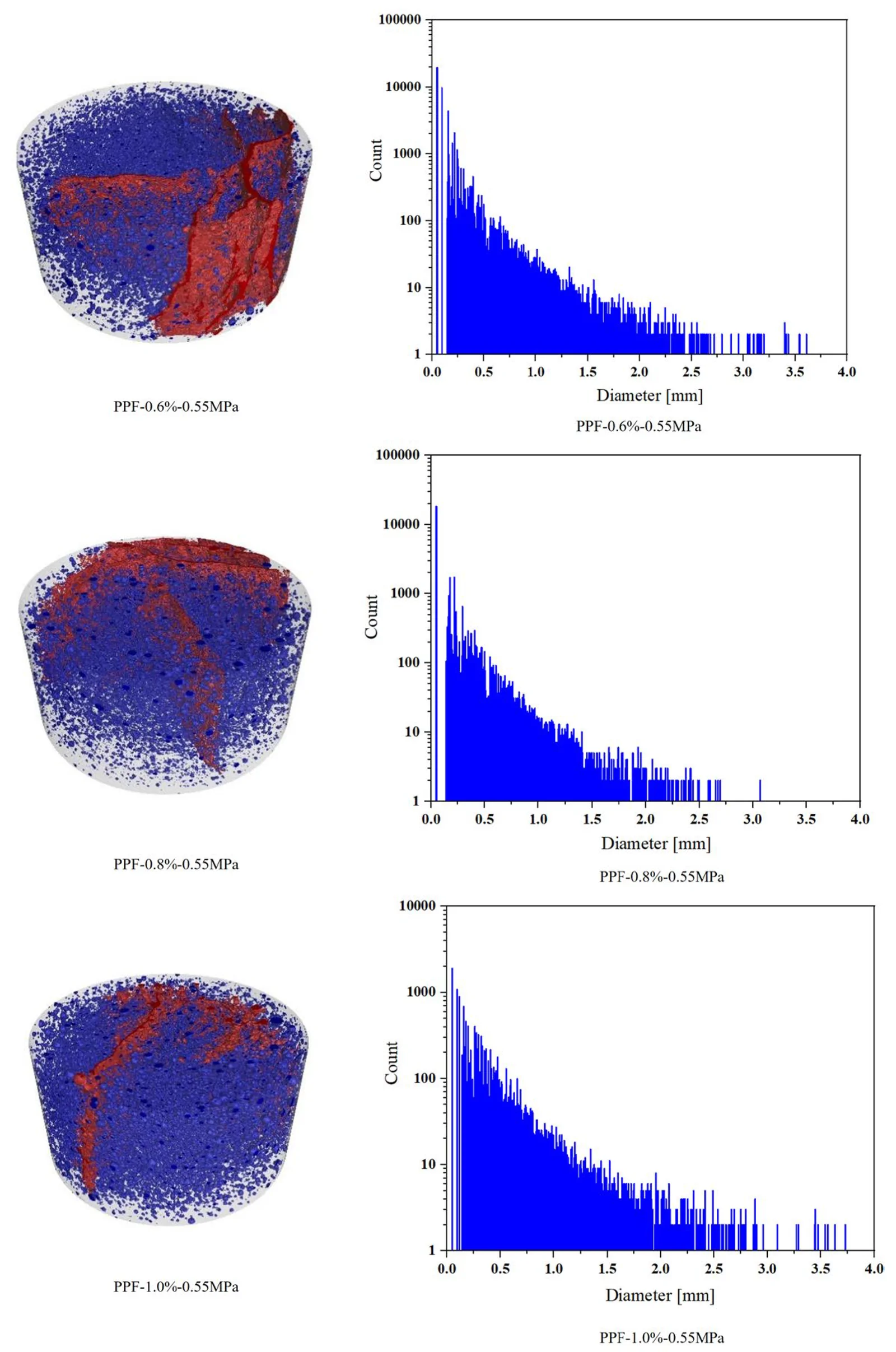
The volume of each detected void against its equivalent radius.

**Table 1:** Mix proportions of mortars (g/kg).

Groups	Slag	Alkali Activator Solution	Sand	PPF Vpf (%)
Ctrl	322.7	177.3	500	0.0
PPF-0.2%	322.7	177.3	500	0.2
PPF-0.4%	322.7	177.3	500	0.4
PPF-0.6%	322.7	177.3	500	0.6
PPF-0.8%	322.7	177.3	500	0.8
PPF-1.0%	322.7	177.3	500	1.0

**Table 2:** Chemical compositions of GBFS.

Materials	Chemical Composition (wt.%)						
	SiO_2_	Al_2_O_3_	CaO	MgO	Fe_2_O_3_	Na_2_O	SO_3_
GBFS	27.3	15.4	44.9	7.9	0.3	0.3	2.3

**Table 3:** Mechanical properties of geopolymer mortar.

Sample	Impact Air Pressure (MPa)	Average Strain Rate /(s^−1^)	Peak Stress /MPa	Ultimate Toughness /(J·cm^−3^)	Peak Strain /10^3^	DIF
PPF-0.0%-1	0.35	48.34	26.14	0.129	5.84	0.815
PPF-0.0%-2	0.45	64.24	29.13	0.190	6.20	0.908
PPF-0.0%-3	0.55	75.61	33.66	0.241	7.24	1.049
PPF-0.0%-4	0.65	81.06	37.53	0.283	7.83	1.170
PPF-0.2%-1	0.35	55.20	28.20	0.161	5.87	0.904
PPF-0.2%-2	0.45	65.42	32.64	0.242	6.47	1.046
PPF-0.2%-3	0.55	80.65	38.37	0.267	7.73	1.229
PPF-0.2%-4	0.65	92.35	43.85	0.276	8.26	1.405
PPF-0.4%-1	0.35	53.91	31.99	0.200	6.66	1.053
PPF-0.4%-2	0.45	70.18	38.01	0.253	6.96	1.251
PPF-0.4%-3	0.55	91.50	40.11	0.310	8.04	1.320
PPF-0.4%-4	0.65	100.83	47.56	0.316	8.86	1.565
PPF-0.6%-1	0.35	59.71	38.51	0.228	6.71	1.206
PPF-0.6%-2	0.45	77.49	43.85	0.275	7.88	1.373
PPF-0.6%-3	0.55	96.15	48.67	0.364	8.45	1.524
PPF-0.6%-4	0.65	109.68	52.16	0.372	9.01	1.633
PPF-0.8%-1	0.35	65.68	43.97	0.209	6.98	1.458
PPF-0.8%-2	0.45	82.42	47.16	0.345	8.11	1.525
PPF-0.8%-3	0.55	101.54	52.46	0.381	9.19	1.697
PPF-0.8%-4	0.65	116.35	56.80	0.469	10.01	1.837
PPF-1.0%-1	0.35	73.94	49.19	0.355	8.41	1.633
PPF-1.0%-2	0.45	94.19	56.29	0.437	9.26	1.869
PPF-1.0%-3	0.55	106.01	60.65	0.498	10.61	1.962
PPF-1.0%-4	0.65	124.78	64.15	0.570	11.13	2.129

## Data Availability

The original contributions presented in this study are included in the article. Further inquiries can be directed to the corresponding author.
